# Shining Light on a Dark Corner: A Case Report on Perianal Basal Cell Carcinoma

**DOI:** 10.7759/cureus.61274

**Published:** 2024-05-28

**Authors:** Soumiya Samba, Ahmed BenSghier, Meriem Bouabid, Soufiane Berhili, Mohamed Moukhlissi, Loubna Mezouar

**Affiliations:** 1 Department of Radiation Oncology, Mohammed VI University Hospital, Faculty of Medicine and Pharmacy, Mohammed First University, Oujda, MAR

**Keywords:** genital, radiation therapy, cancer, ulcer, perianal region, lesion, basal cell carcinoma

## Abstract

Perianal basal cell carcinoma (BCC) is a rare occurrence, with limited documented cases in the existing literature. The precise etiology contributing to its onset remains inadequately elucidated. Macroscopically, perianal BCC often exhibits atypical features, potentially leading to diagnostic challenges. Histopathological examination plays a crucial role in distinguishing BCC from other cutaneous lesions in this region. Despite its localized nature, perianal BCC generally carries a favorable prognosis characterized by a gradual progression. However, diligent follow-up is essential to mitigate the risk of recurrence.

Our clinical observation highlights a noteworthy yet uncommon manifestation. The patient, a 64-year-old male, presented with a persistent perianal lesion evolving over a three-month period. Symptoms included intermittent bleeding and purulent discharge, exacerbating the clinical picture. A biopsy was subsequently performed, confirming the diagnosis of basal cell carcinoma. Following this, the patient underwent external beam radiation therapy as part of the treatment regimen.

## Introduction

Basal cell carcinomas (BCCs) represent the most prevalent malignancies affecting the skin, surpassing the incidence of malignancies in any other specific tissue or organ, both individually and collectively [1]. Their epidemiology and etiology have been extensively studied, revealing a multifaceted interplay of genetic predisposition, environmental factors, and ultraviolet (UV) radiation exposure [2,3].

Geographical regions characterized by heightened and prolonged exposure to sunlight have consistently shown elevated rates of BCCs, underlining the significant role of UV radiation in their pathogenesis [2,4]. Furthermore, individuals with fair skin lacking the protective attribute of melanin are particularly susceptible to this type of skin cancer, resulting in approximately 85% of BCCs manifesting in the facial and cervical regions [5].

While the association between UV exposure and BCC development is well-established, intriguing variations in tumor distribution have been observed. Occurrences in areas devoid of ultraviolet exposure, notably the genital and perianal regions, are exceedingly rare but not unprecedented [3,5]. Despite their rarity, BCCs in these anatomical sites present unique challenges in diagnosis and management, often requiring a multidisciplinary approach for optimal care [2,3].

In this context, our case report sheds light on a rare presentation of basal cell carcinoma in the perianal region, emphasizing the importance of vigilance in recognizing atypical manifestations of this common malignancy. By documenting and analyzing such cases, we contribute to the expanding body of literature aimed at elucidating the full spectrum of BCC presentations and refining diagnostic and therapeutic strategies.

## Case presentation

We present the case of a 64-year-old male, employed as a professional worker, who had an unremarkable personal and family medical history, notably devoid of anogenital warts, sexually transmitted diseases, malignancies, inflammatory dermatoses, or exposure to arsenic. He presented with a perianal lesion that had progressively enlarged over a period exceeding three months. Symptoms emerged within the context of overall well-being. Clinical examination revealed a red-colored perianal lesion with infiltration extending toward the anal border on the right side. No palpable inguinal lymphadenopathy or other visible skin abnormalities were noted. Proctological examination identified a raised lesion localized on the right side.

**Figure 1:**
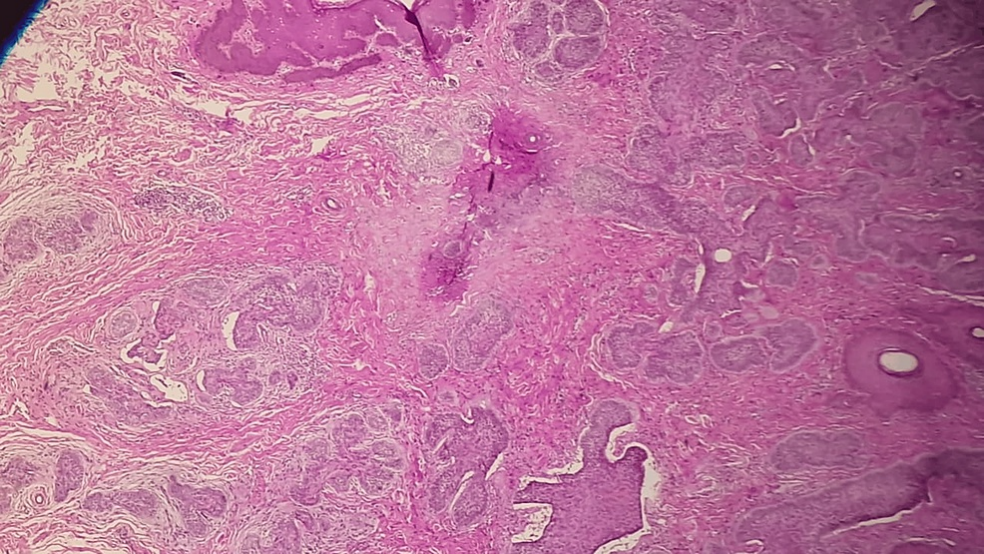
Histopathology image Histological image showing tumoral proliferation arranged in clusters. Tumor cells are bluish and form a peripheral palisade. Retraction spaces are also observed Between the Proliferation and the Stroma (Hematoxylin and Eosin, 10X).

Histopathological analysis of a punch biopsy obtained from the lesion demonstrated an oedematous stroma containing mononuclear inflammatory cells beneath a stratified squamous epithelium. Pleomorphic cells within epithelial cell islands displayed infiltrative growth, along with tumor cell islands exhibiting peripheral palisading which leads to basal cell carcinoma (Figure 1).

**Figure 2:**
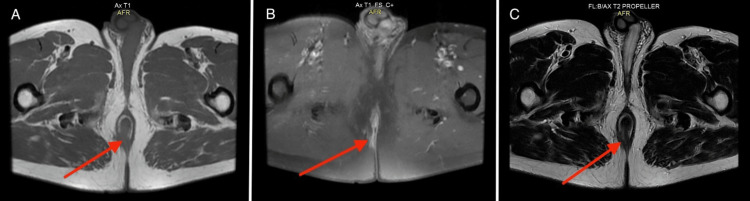
Pelvic MRI with contrast agent injection (A) Axial section T1 sequence (B) Axial section T1 sequence with fat saturation (C) Axial section T2 sequence. The images are revealing a tumoral process (red arrows) in the perianal region. The parasagittal median has well-defined regular contours, showing Hyposignal T1 and Hypersignal T2, enhancing post-contrast injection.

Subsequent pelvic magnetic resonance imaging (MRI) revealed a tissue abnormality measuring 26 × 12 mm at the right lateral anal orifice. This anomaly demonstrated involvement of the internal and external sphincter muscles, with discrete infiltration of pelvic rectal fat (Figure 2).

**Figure 3:**
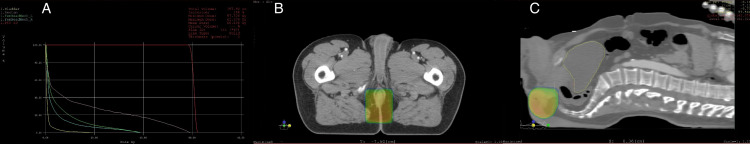
Radiotherapy images (A) Radiotherapy was administered following the recommended doses for the organs at risk, (B) Axial section, (C) Sagittal section, and the dosimetric planification of radiotherapy colorwash dose coverage of the perianal tumor.

The patient underwent radiation therapy, receiving a total dose of 60 Grays (Gy) in 30 daily fractions (2 Gy/fraction, 5 days/7) to the primary tumor planning target volume (PTV), the GTV was identified by the MRI (clincal tumoral volume (CTV) = gross tumoral volume (GTV) + a 1 cm margin), while the organs at risk (OAR) were rectum, bladder and both of femoral heads, with a dose respectively of V60< 50%, V60<50%, V50<10%. A three-dimensional conformal technique employing 6 Megavolt photon beam radiotherapy was utilized in a dorsal position (Figure 3).

**Figure 4:**
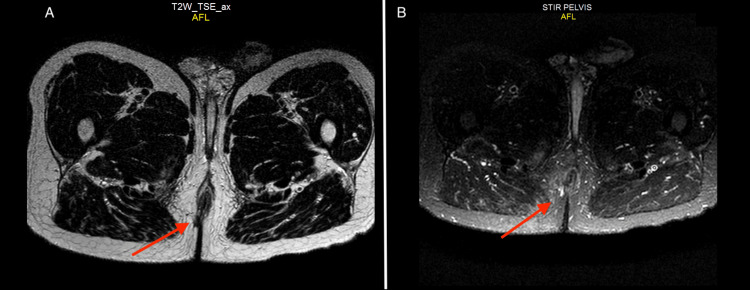
Post-treatment pelvic MRI (A) Axial section T2 sequence, (B) Axial section T2-STIR sequence demonstrating disappearance of the perianal process (red arrows), suggestive of complete tumor response. STIR: Short tau inversion recovery

Radiation therapy was administered without significant acute, sub-acute, or chronic side effects. Three months post-treatment completion, a comprehensive assessment was conducted, including clinical examination and pelvic MRI. Results indicated the absence of residual tumor cells or remnants (Figure 4).

## Discussion

Basal cell carcinoma (BCC) stands out as the predominant malignancy prevalent in the human population, primarily manifesting in sun-exposed areas like the head and neck region. However, occurrences of basal cell carcinomas (BCCs) in the perianal area constitute a rare subset, comprising less than 0.2% of all anorectal malignancies. Existing literature primarily comprises case reports or brief series documenting such instances [1,2].

The emergence of basal cell carcinoma (BCC) in sun-shielded body regions suggests the involvement of other causative factors besides UV radiation. Several extensive cohort and case-control studies have linked certain lifestyle choices with an increased propensity for basal cell carcinoma (BCC) development. Notably, evidence indicates a modest yet statistically significant elevation in BCC likelihood among alcohol users, while both cigarette smoking and obesity seemingly reduce BCC incidence [3,4]. Additionally, a high prevalence of trauma, past radiation exposure, and other skin lesions have been observed in conjunction with BCC [5].

Although human papillomavirus (HPV) positivity and p16INK4a upregulation may occur in anal squamous cell carcinomas, HPV is not implicated in perianal BCC etiopathogenesis [6]. Diagnosis of perianal BCC may be indicated by characteristic palisading appearance and Ber-EP4 monoclonal antibody expression [7]. Differential diagnosis includes external hemorrhoids, anal cysts, Bowen's disease, condyloma acuminatum, Paget's disease, and basaloid squamous cell carcinoma (BSCC) [8], the latter requiring distinct treatment approaches due to its higher metastatic risk [6].

Delayed diagnosis is frequently observed, attributable to various factors such as patients' reluctance to seek medical evaluation for perceived minor symptoms, misdiagnosis of BCC as other skin lesions, the rarity of BCC in sun-protected areas, diverse macroscopic appearances of BCC resembling common perianal lesions, and pigmentation in BCC of dark-skinned individuals often mistaken for malignant melanoma [6,7].

Treatment options include local excision or radiotherapy, with local excision proving curative in up to 95% of cases, and local recurrence being uncommon [7,8]. Successful radiotherapy treatment has been reported for patients unfit for surgery [9]. The recurrence rate for adequately resected perianal BCC appears minimal, as reported by Gibson et al. [6]. Long-term follow-up involves surveillance for BCC in other locations, given individuals with one BCC instance face up to a 50% risk of subsequent primary BCC within five years. However, it remains unclear if this risk is comparable between trauma-related and sunlight-exposed BCC [10].

## Conclusions

Cases of basal cell carcinoma (BCC) situated in the perianal region frequently present at an advanced stage compared to other BCC variants. Such delayed diagnoses may lead to advanced disease of these lesions as inflammatory or infectious, potentially leading to erroneous treatment modalities. Therefore, the histopathological evaluation of perianal lesions assumes paramount significance. It is noteworthy to emphasize the utilization of local excision as a therapeutic intervention is the best option in the first stages, subsequent to a proper diagnosis, and has shown to be efficacious in achieving a cure without the need for more advanced interventions.
